# A pilot qualitative study of patient understanding and perceptions of genetic counselors and cascade testing in the context of transthyretin cardiac amyloidosis

**DOI:** 10.1002/jgc4.70249

**Published:** 2026-06-21

**Authors:** Quan M. Bui, Taylor Berninger, Ana Morales, Lauren Korty, Larry A. Allen, Eric D. Adler, Marcus A. Urey, Kimberly N. Hong, Borsika Rabin, Cinnamon Bloss

**Affiliations:** ^1^ Division of Cardiovascular Medicine, Department of Medicine University of California, San Diego La Jolla California USA; ^2^ Altman Clinical and Translational Research Institute Dissemination and Implementation Science Center University of California, San Diego La Jolla California USA; ^3^ Center for Empathy and Technology, T. Denny Sanford Institute for Empathy and Compassion University of California, San Diego La Jolla California USA; ^4^ Augustana‐Sanford Genetic Counseling Graduate Program Augustana University Sioux Falls South Dakota USA; ^5^ Department of Genomic Health Geisinger Health Danville Pennsylvania USA; ^6^ Division of Genetic Counseling University of California, San Diego La Jolla California USA; ^7^ Division of Cardiology, Department of Medicine University of Colorado School of Medicine Aurora Colorado USA; ^8^ Herbert Wertheim School of Public Health and Human Longevity Science University of California, San Diego La Jolla California USA; ^9^ Department of Psychiatry University of California, San Diego La Jolla California USA

**Keywords:** cardiac amyloidosis, cardiomyopathy, genetic counselor, genetic testing, heart failure, implementation science, informed consent

## Abstract

Genetics‐informed care is becoming increasingly important in the management of cardiomyopathy (CM). Despite current guidelines recommending genetic testing and counselors, these services are vastly underutilized. To our knowledge, this is the first qualitative study to examine patient perspectives (*n* = 10) on genetic counselors and cascade testing in the context of cardiovascular care in transthyretin amyloidosis (ATTR), with the goal to inform implementation strategies for integrating genetic services. Codebook thematic analysis of semistructured interviews was conducted with adult patients diagnosed with ATTR or at‐risk family members between June and August 2024. Data were evaluated through deductive and inductive coding. Five key themes were identified: (1) patients had limited understanding of genetic counselors' roles; (2) the likelihood of a patient seeing a genetic counselor was significantly influenced by strength of cardiovascular provider recommendation; (3) patient experiences with genetic counselors were generally favorable, especially psychosocial support; (4) patients suggested additional refinements for genetic counseling, including the desire for cardiac‐specific expertise; (5) patients faced considerable challenges communicating the importance of genetic testing to family members, largely due to stigma and family members' preference to avoid distress associated with testing. These findings highlight the need for implementation of science‐based approaches to improve patient education about genetic counselors, emphasize recommendations from cardiovascular providers, train cardiac‐specific genetic counselors, and develop innovative tools to support family communication around cascade testing. Addressing these areas can enhance the integration of genetic services into routine CM care.

**What is known?**

Genetics‐informed care is increasingly relevant in cardiovascular medicine, particularly for inherited cardiomyopathies such as transthyretin amyloidosis. Despite guideline recommendations, integration of genetic testing, genetic counseling, and cascade testing into routine cardiovascular practice remains inconsistent and often limited.

**What does this paper add?**

Provides in‐depth qualitative insights into patient perspectives on genetic counselors and cascade testing in the context of transthyretin amyloidosis. Identifies key patient‐reported barriers to genetics‐informed care: (1) Limited understanding of the role of genetic counselors, (2) Strong dependence on cardiovascular provider recommendation to facilitate genetic counselor referrals, (3) Overall positive experiences with genetic counselors, though cardiac‐specific expertise was desired, (4) Frustration when communicating genetic risk and cascade testing to family members.

## 1 INTRODUCTION

Genetics‐informed care has become increasingly important in the management of cardiomyopathy (CM), including transthyretin amyloidosis (ATTR) (Hershberger et al., 2018). ATTR‐related CM is characterized by left ventricular hypertrophy, restrictive cardiomyopathy, and heart failure (Writing Committee et al., 2023). In hereditary or variant cases (ATTRv), these cardiac manifestations are often accompanied by extracardiac features, such as peripheral neuropathy and autonomic dysfunction. ATTRv results from pathogenic variants in the *TTR* gene that are inherited in an autosomal dominant manner (Writing Committee et al., 2023). Therefore, a molecular diagnosis has important clinical implications for cascade testing of first‐degree relatives, who have a 50% risk of inheriting a clinically significant variant, although disease expression is complicated by variable expression and incomplete penetrance. Accordingly, *TTR* genetic testing and counseling are recommended for all patients with ATTR (Writing Committee et al., 2023).

The recent surge in ATTR treatments and clinical trials has increased awareness of opportunities to improve patient identification, including emphasis on genetic evaluation strategies. However, studies suggest that genetics‐informed care in CM, including genetic testing and cascade testing, remains underutilized (Cirino et al., 2022; Morales et al., 2024; Tiner et al., 2022). For example, retrospective analyses of insurance claims and electronic medical records estimate that only 1% of patients with CM underwent genetic testing (Bui et al., 2026; Morales et al., 2024). While genetic testing rates in ATTR are likely higher than in CM overall due to therapeutic implications, available data remain limited (Bui et al., 2026). One study using the Veradigm Health Database reported that genetic testing was performed in approximately 6% of patients with ATTR (Longoni et al., 2023). According to a 2024 survey by the Amyloidosis Research Consortium, approximately 69% of patients with ATTR underwent genetic testing before diagnosis (Amyloidosis Research Consortium, 2024). Barriers to broader implementation of genetics‐informed care in CM include inadequate cardiovascular provider knowledge, complex clinical workflows, and limited access to genetic specialists (Schaibley et al., 2022).

Given cardiologists' challenges in meeting this demand, successful genetics‐informed care increasingly depends on genetic specialists and innovative implementation strategies (Hull et al., 2025; Morales et al., 2023). Genetic counselors, trained in genetic risk assessment and the psychosocial and familial implications of inherited diseases, are essential to delivering genetics‐informed care (Arscott et al., 2016; Moharem‐Elgamal et al., 2020). Genetic counselors collaborate with cardiologists, neurologists, and gastroenterologists to provide comprehensive ATTR and CM care by facilitating genetic testing, communicating results, as well as providing risk counseling and psychosocial support (Brito et al., 2023; Bui et al., 2025). Patients receiving care from genetic counselors report improved knowledge, understanding, and satisfaction (Armstrong et al., 2015). Nonetheless, genetic counselors are underutilized in cardiology, with only 0.75% of individuals with a cardiovascular genetic condition receiving genetic counseling (Hellwig et al., 2024).

Patient perspectives are essential to understanding underutilization of genetic counselors, yet patient‐specific perceptions of these services in ATTR remain poorly understood (Hendricks‐Sturrup et al., 2023). While prior qualitative work has examined patient perspectives on genetic counselors in other inherited conditions, to our knowledge, this is the first such pilot study conducted in the context of cardiovascular care in ATTR. Semistructured interviews are uniquely positioned to explore disease‐specific themes not accessible through surveys or quantitative analyses, with findings intended to directly inform implementation strategies for integrating genetic services into routine cardiovascular care in ATTR.

## 2 METHODS

This single‐center qualitative study included patient needs assessment interviews conducted between June and August 2024. A qualitative study design was used to reveal themes and generate assertions that facilitate an in‐depth understanding of patient perspectives (Creswell, 2013). The study was overseen by a multidisciplinary research team with expertise in advanced heart failure, cardiac amyloidosis, genetics, genetic counseling, qualitative methodology, and implementation science. The study received appropriate ethical oversight and was approved by the institutional review board, which determined that the study was exempt under category 45 CFR 46.104(d).

Participants were recruited from the UC San Diego Cardiac Amyloidosis Clinic through two approaches: (1) direct referral from treating heart failure cardiologists (Q.B. and M.U.), and (2) outreach via telephone by the study team to eligible patients and at‐risk family members identified during routine clinical care. We used purposive sampling to ensure representation across key demographic characteristics. Sampling decisions were made iteratively, with attention to sex assigned at birth (or gender, when documented) and self‐reported ancestry. Representativeness was assessed by comparing the sample characteristics to those of the broader population within our health system. Although formal demographic data were collected at the beginning of each interview, the study team had access to basic demographic data (e.g., race, sex) via the electronic medical record, which informed our sampling strategy. Inclusion criteria included adults (≥18 years) who were either diagnosed with ATTR or were first‐degree relatives of affected individuals, English‐speaking, and willing to participate in a recorded interview. Exclusion criteria included cognitive impairment that limited the ability to consent or participate in interviews. At our institution, genetic counselor referrals are made at the discretion of the treating cardiologist. Typical indications for referral include support for informed decision‐making before testing, assistance with result interpretation, and guidance around cascade testing for at‐risk family members. Genetic counseling is available both in‐person and via telehealth. It is important to note that cardiology shares a single genetic counselor with the prenatal genetic service, which limits availability. All participants provided informed consent before participation in this study.

Interview guides were developed based on discussions with the core research team and were iteratively refined. The interview included various questions organized by three domains (Patient Disease History, Genetic Testing and Counseling, and Health Information Delivery), which were chosen based on prior clinical experience and the components of a genetic evaluation for cardiomyopathy (Hershberger et al., 2018). The interview guide is included in Appendix 1. In addition to the semistructured interview questions, participants were asked about demographic characteristics (i.e., self‐identified race, highest level of education, occupation, health insurance, etc.). Interviews were conducted and recorded using Zoom by the lead author (Q.B.) who is an expert in the content and discussion topics. No one else was present for the interviews besides Q.B. and the participants. Repeat interviews were not conducted. Participants did not receive monetary compensation for their participation. Field notes were completed after the interviews. These notes were incorporated into the coding process and team discussions to identify potential inductive codes, revise the codebook, and develop preliminary themes.

Recorded interviews were transcribed professionally using GoTranscript services and were verified for accuracy by the lead author (Q.B.). All raw data were deidentified, and participants were assigned unique code numbers to ensure confidentiality. Transcripts were not returned to the participants for comments or corrections. We conducted a codebook thematic analysis, combining deductive and inductive coding (Braun & Clarke, 2020). Initial codes were developed deductively, based on the interview guide and prior clinical experiences related to genetic testing and counseling in cardiomyopathy patients. Codes reflected concepts such as “delays in medical care,” “decision to accept genetic testing,” “risks of genetic testing,” “importance of a genetic counselor,” “informed consent,” etc. However, the analysis remained open to inductive insights that were identified from participant narratives. This process was conducted with attention to the study aims and not privileging deductive over inductive strategies. Specifically, codes that did not seem to fit the planned deductive analysis were flagged for team discussion and for monitoring whether they appeared in other transcripts frequently enough to warrant inclusion as an inductive code. Team discussions were held to decide which inductive codes could be added and how to iterate on the codebook. Whenever codes were added, all transcripts were subsequently recoded with an amended codebook. We managed both deductive and inductive approaches by simultaneous coding with attention to the study aims and not privileging one over the other. A final interpretation of the data was conducted to integrate these approaches, allowing a comprehensive examination of the data. This approach allowed us to examine anticipated barriers while remaining open to novel perspectives. Our analysis was grounded in the post‐positivist paradigm, with the aim of identifying shared experiences while acknowledging the influence of the research context (Cornish & Gillespie, 2009). A subset (30%) of the transcripts was coded together by both the lead author (Q.B.) and co‐author (B.R.) until consensus was reached, revising code applications as needed; no additional codes were added through this process. This collaborative coding was conducted during the early stages of analysis, in real time, for analytic alignment and codebook refinement. The remaining transcripts were coded by the lead author (Q.B.). Theoretical sufficiency was used to guide sample size determination and was assessed through iterative analysis (Saunders et al., 2018). Opportunities for theme development, through increasing depth and breadth to address the research question, were not identified after coding the seventh transcript. The final three transcripts were used to confirm the stability of the thematic framework. Qualitative data were managed using the qualitative software package, ATLAS.ti v.23.0 (Scientific Software Development GmbH, Berlin, Germany).

### 2.1 Researcher positionality

The lead interviewer and primary coder (Q.B.) is a male, advanced heart failure and transplant cardiologist with clinical expertise in cardiac amyloidosis and genetic cardiomyopathy. His clinical role and familiarity with some of the participants may have influenced the interview dynamics, including participant comfort or willingness to disclose information. To minimize bias, interviews were designed to be participant‐led, using open‐ended prompts and reflective probing. A second coder (B.R.), a qualitative methodologist with no clinical role or prior relationship with the participants, contributed as an outsider perspective to the data analysis. This interdisciplinary coding process enabled triangulation and reflexivity, integrating both clinical contextual knowledge and methodological rigor. Reflexivity in our post‐positive paradigm particularly reflects our responsibility to understand our participants. We recognize that thematic analysis is inherently shaped by researcher subjectivity. Practicing reflexivity through critical self‐reflection allowed us to examine bias and reduce the possibility of making assumptions not supported by the data. We distinguish our practice of reflexivity from that used in interpretivist and constructivist paradigms, which acknowledge the researcher's role in shaping meaning.

## 3 RESULTS

A total of 10 participants were interviewed, including 6 individuals with a diagnosis of ATTR (probands) and 4 at‐risk family members. Participants had a median age of 69.5 years and were predominantly male (80%) and White (70%) (Table 1). All participants had genetic testing completed and only 30% had met with a genetic counselor. The frequency of TTR genetic variants was as follows: none (3), Y116S (p.Y136S) (1), V122I (p.V142I) (2), T60A (p.T80A) (1), V30M (p.V50M) (1), N38A (p.N58A) (1), and F64L (p.F84L) (1). Of note, all participants approached for the study agreed to proceed. Interviews lasted between 35 and 65 min. Five primary themes were identified from the patient interviews regarding genetic counselors and cascade testing: (1) patients had limited understanding of genetic counselors' roles; (2) the likelihood of a patient seeing a genetic counselor was significantly influenced by strength of cardiovascular provider recommendation; (3) patient experiences with genetic counselors were generally favorable, especially psychosocial support; (4) patients suggested additional refinements for genetic counseling, including the desire for cardiac‐specific expertise; (5) patients faced considerable challenges communicating the importance of genetic testing to family members, largely due to stigma and family members' preference to avoid distress associated with testing.

**TABLE 1:** Participant demographics.

	Participants (*n* = 10)
Age, years (IQR)	69.5 (50–71)
Sex, *n* (%)	
Male	8 (80)
Female	2 (20)
Race, *n* (%)	
White	7 (70)
Black	2 (20)
Asian	1 (10)
Hispanic	0 (0)
Education, *n* (%)	
High school	1 (10)
Associate	3 (30)
Bachelors	5 (50)
Graduate	1 (10)
Insurance, *n* (%)	
Medicare/Medi‐Cal	5 (50)
Private	4 (40)
None	1 (10)
Genetic characteristics, *n* (%)	
Proband	6 (60)
At‐risk family member	4 (40)
Genetic counselor seen	3 (30)
Genetic test positive	7 (70)

## 4 LIMITED UNDERSTANDING OF GENETIC COUNSELORS' ROLES

Generally, patients did not understand the role of the genetic counselor within their medical team, often describing their responsibilities incorrectly. Some assumed genetic counselors primarily provided emotional support for distressing news, rather than guidance on how to manage genetic conditions, including interpretation and implication of results pertaining to clinical disease.

I never thought about what a genetic counselor does because I didn't know there was such a thing as a genetic counselor. Participant 9 (proband)

I'm assuming a genetic counselor is someone who explains genetics to you and how it helps or hurts your situation. That's just an assumption. What do they do? Participant 9 (proband)

Based on the name, he or she does something with genetics. What is he or she counseling me about? Amyloidosis? Participant 1 (proband)

Saying that if you know about the genetic test results, it may hurt you emotionally or whatever. That's what a counselor does, is deal with your thoughts about things. Participant 8 (proband)

These misunderstandings may reflect how genetic counselors are framed within cardiology clinics. Due to lack of access to genetic services, cardiology providers often must deliver genetics‐informed care themselves, unlike oncology or prenatal care, where genetic counseling is more routine. This gap in framing limits patient awareness and ultimate use of genetic counselor services.

## 5 SEEING A GENETIC COUNSELOR INFLUENCED BY THE STRENGTH OF CARDIOVASCULAR PROVIDER RECOMMENDATION

Emphasized recommendations from trusted cardiovascular providers greatly increased patient acceptance of genetic counseling. Patients considered their cardiovascular provider's advice decisive and suggested integrating genetic counselors clearly into standard multidisciplinary care plans.

If my doctor told me to do it, I would do it. I have done a lot of reading about him and medical therapies, so I felt pretty confident I was in the right hands. I know he's a leader in cardio ATTR. Participant 6 (family member)

I don't remember exactly, but after we were diagnosed, it was like, ‘Well, now that you're diagnosed, you should see a neurologist and you should see a gastroenterologist.’… We were automatically given a consult with the neurologist and the gastroenterologist. If you guys had an in‐house genetic counselor in the clinic, then it would be like, ‘Well, now that you're diagnosed, you have a consult to genetic counselor’. Participant 3 (family member)

These findings highlight how deeply cardiology providers shape patient engagement with genetics. In the absence of clear institutional workflows and limited access to genetic counselors, the cardiology provider functions as the gatekeeper to genetic services.

## 6 PATIENT EXPERIENCES WITH GENETIC COUNSELORS WERE GENERALLY FAVORABLE, ESPECIALLY PSYCHOSOCIAL SUPPORT

Patients reported that their experiences with genetic counseling exceeded their initial expectations, particularly in terms of the professionalism and thoroughness of care provided. Many patients anticipated a quick and impersonal process but were instead met with a structured, informative, and supportive experience. Some patients expressed that their non‐genetic healthcare providers did not provide adequate emotional support compared with genetic counselors. Also, they particularly valued the genetic counselor's approach, which balanced warmth and professionalism. Instead of focusing on sympathy, genetic counselors provided psychosocial support with an actionable perspective, which patients found reassuring and empowering. Given the small number of participants who had received genetic counseling (30%), formal subgroup comparisons stratified by prior genetic counseling exposure were not performed.

I had this idea that I would just sort of bop into a place, they'd take a little blood, I'd bop out, and they'd call me with the results later. It was much more organized and serious than I thought it would be, which is good because I ended up being positive…I'm glad the genetic counselor treated me the way that she did.’ At the time I was like, ‘For sure, it's fine. I'm going to be fine. I totally don't have the gene. What are the odds? Participant 7 (family member)

A lot of healthcare providers don't understand from a human standpoint, how it (genetic test results) can weigh on someone. I think that's lacking with a lot of healthcare providers that there's just other things to consider other than like, your test was positive. Participant 3 (family member)

My genetic counselor wasn't particularly like, ‘Oh, I'm so sorry.’ This like pitying thing. It was like, ‘This is what's happening.’ Just very professional, but also warm. It's a hard line to walk. Participant 7 (family member)

Genetic counselors helped patients navigate disease management as well as facilitate referrals to specialists. Genetic counselors effectively supported patients in cascade testing decisions, particularly concerning minors. In current practice, routine predictive genetic testing for TTR variants in asymptomatic minors is generally not recommended because of the adult‐onset nature of ATTR and the absence of interventions that would change management.

My genetic counselor knew a lot about amyloidosis. I already had known some of it, just because we all started talking, and researching, and reading about it before the test. But it was still very good that my genetic counselor was very prepared…‘This is what the standard of care is. This is what you probably want to consider doing at some point in the future.’ Participant 7 (family member)

As soon as I found out I was positive, I wanted to get my son tested, but then my doctor set up an appointment with a genetic counselor, and then we discussed like the pros and cons of getting him tested, and I decided to hold off… I felt like the genetic counselor definitely helped me to widen my perspective when it came to testing my son. In my mind, I was like, why wouldn't I get my son tested? I want to know as soon as possible if he has this mutation or possibility of having amyloidosis in the future. It wasn't until after talking to her and her presenting all these other facets of it that I made the decision not to get him tested. Participant 3 (family member)

My genetic counselor put the bug in my ear that, ‘You should think about having some baseline evaluation testing done for your heart, and all of this stuff, and your nerves, and make sure that we know where you're at.’ Participant 7 (family member)

## 7 ADDITIONAL REFINEMENTS FOR GENETIC COUNSELING

Patients expressed appreciation and value of genetic counselors, but also emphasized a need for disease‐specific knowledge. ATTR is a multisystem disease involving cardiac, neurologic, and gastrointestinal manifestations, thus requiring nuanced understanding across domains. When counselors lacked cardiac‐specific knowledge, participants noted confusion or perceived irrelevance of counseling content. This underscores the need for genetic counselors with cardiac expertise and greater interdisciplinary collaboration with knowledge sharing.

My genetic counselor went overboard and was going into all the different types of amyloidosis because she didn't know the specific type. My doctor whittled that down himself. The genetic counselor covered everything liver and joints. She was covering it all and I'm like, ‘I don't have that. I got the neuropathy and maybe the heart thing.’ Participant 6 (family member)

Patients found that genetic counselors went beyond genetic testing results and medical implications. Specifically, patients valued genetic counselors' attention to psychosocial factors that were often not covered by their cardiovascular providers. Patients preferred in‐person discussions for delivering positive genetic testing results.

From a medical standpoint or the clinical side of it, my doctor does a really, really great job. A lot of healthcare providers don't understand from a human standpoint how it can weigh on someone and that sort of thing. I think that's something that's lacking with a lot of healthcare providers. There are just other things to consider other than like, your test was positive… My doctor is great at the medical side of it, educating on disease progression and that sort of thing. Again, the more psychological aspects and different things that we should consider, the genetic counselor had a lot more understanding or knowledge on that. Participant 3 (family member)

I really had a lot of respect for the genetic counselor. I should have known something was up when she told me to come into the office, because I thought it was still nothing. Then I sat down and she told me, and I was like, ‘Oh, actually, it's kind of good that I was actually here.’ This would have been weird to have received the news over the phone. Participant 7 (family member)

## 8 PATIENTS FACED CHALLENGES WHEN COMMUNICATING THE IMPORTANCE OF CASCADE GENETIC TESTING TO FAMILY MEMBERS

Patients expressed significant frustration in educating family members about the disease and the importance of genetic testing. Stigma associated with the disease led family members, particularly asymptomatic adults, to adopt an avoidance approach toward genetic testing. These findings suggest that successful cascade testing requires not only education but also emotionally attuned, culturally sensitive communication strategies.

You don't want to go out, tell everybody you got herpes or something. People look at disease as a defect and they don't want to be judged so they don't want to tell anybody about any medical issues. Participant 6 (family member)

For adult children, it was baffling to me when the children of patients chose not to be tested. That ignorance is bliss kind of thing. I don't have an official diagnosis so I can ignore that fact. Participant 3 (family member)

Sometimes it's not easy to get family to test, especially when it's something they don't know about and it scares the heck out of them to even talk about it. Participant 5 (proband)

I told my kids that they should get tested. It was like they would rather not know. Grown folk now. I don't try to interfere with grown folk's decisions anymore. Participant 5 (proband)

I don't talk to my cousins much anymore. I don't know if they've been tested. A lot of them don't really want to talk about it, because they don't have symptoms. Participant 7 (family member)

## 9 DISCUSSION

The purpose of this pilot study was to provide an in‐depth understanding of the perspectives on genetic counselors and cascade testing among patients with ATTR and at‐risk family members who were seen at the University of California San Diego amyloidosis clinic. While prior qualitative work has examined patient perspectives on genetic counselors in other inherited conditions, to our knowledge, this represents the first such investigation in the context of cardiovascular care in ATTR, addressing a critical gap in the evidence base for implementation science‐based strategies in genetics‐informed cardiovascular care.

Genetic counselors are critical to genetics‐informed CM care but remain underutilized due to limited availability and inadequate integration into clinical workflows (Hellwig et al., 2024; Morales et al., 2023). However, studies suggest that genetic counselors are underutilized even when available, suggesting other barriers are present (Statement AP, 2020). Our results revealed that patients often misunderstood the role of genetic counselors, with some interpreting “counseling” as limited to emotional support rather than a component of comprehensive genetic management. While most of the participants in our study have never received genetic counseling, this lack of experience reflects real‐world care in ATTR and offers critical insights into patient perspectives before genetic counseling engagement. For these participants, perceptions of genetic counselors were likely shaped predominantly by indirect factors, including how genetic testing was framed by cardiovascular providers and broader societal narratives surrounding genetics. Furthermore, a Canadian survey reported similar misconceptions, with over 65% of respondents stating that they had never heard of genetic counseling, with others perceiving that genetic counseling was predominantly for family planning and understanding ancestry (Maio et al., 2013). Such misunderstandings about genetic counselors' scope may contribute to only two‐thirds of referred patients following through with seeing a genetic counselor, underscoring the need for targeted patient education to enhance service utilization (Silver et al., 2024).

Provider recommendation was pivotal in a patient's decision to see a genetic counselor. This finding mirrors evidence from oncology, where a lack of provider recommendation contributes significantly to disparities in access to genetic counseling (Armstrong et al., 2015). Limited availability of genetic counselors with cardiology expertise may further contribute to low provider referral rates. Notably, the 2024 National Society of Genetic Counselors survey found that only 4% of counselors specialized primarily in cardiology (National Society of Genetic Counselors, 2024). In our study, a few participants expressed a preference for cardiac‐focused, disease‐specific information. These findings should be explored in future studies, as they underscore the need to examine pathways for cardiology‐specific training for genetic counselors. Integrating cardiac‐specific genetic counselors into multidisciplinary care models may improve patient engagement, downstream uptake of genetic services and clinical outcomes. Furthermore, the growing number of cardiac conditions (e.g., ATTRv, dilated CM, and arrhythmogenic CM) in the American College of Medical Genetics and Genomics secondary findings list highlights the increasing relevance of cardiology training for genetic counselors (Miller et al., 2022).

Patients frequently struggled with communicating the importance of genetic testing to family members, which limits the effectiveness of cascade screening. Traditional methods relying on patients to inform family members have proven suboptimal, resulting in selective and incomplete family communication (Kinnamon et al., 2023). While predictive genetic testing is a personal decision and the choice to forgo testing is valid, patient narratives in this study suggest that avoidance may be driven by limited access to information and counseling as well as psychosocial concerns (e.g., stigma, fear) rather than informed decision‐making. Although genetic counselors support patients through tools such as family letters, their reach remains limited. Innovative communication approach, like the validated *Family Heart Talk* booklet, demonstrate potential in improving cascade screening among first‐degree relatives (Kinnamon et al., 2023). Future research should prioritize additional innovative approaches that facilitate comprehensive family communication and support both cardiovascular providers and genetic counselors in improving informed decision‐making around cascade testing.

### 9.1 Practice implications

This study sheds light on the underutilization of genetic counselors in cardiology. Several barriers were identified, including limited awareness of the role of genetic counselors, the need for cardiology‐specific expertise, and challenges communicating with relatives about cascade testing. In addition to these patient‐ and provider‐level barriers, structural factors such as billing and reimbursement constraints further limit access to genetic counseling services and contribute to underutilization in routine cardiovascular care, although these factors were not the focus of the present study. These combined barriers represent important departure points for the development of implementation strategies to support the integration of genetic counselors and cascade testing in ATTR care. The insights derived from this work will inform future implementation science studies aimed at designing interventions that facilitate collaboration between cardiologists, genetic counselors, patients, and their at‐risk family members. These interventions may include improved patient education about genetic counselors, emphasized recommendations from cardiovascular providers, training of cardiac‐specific genetic counselors, and the development of innovative tools to support family communication around cascade testing. Future research should also prioritize understanding the barriers and facilitators to genetic disclosure and cascade testing among Black families. In the United States, this is particularly relevant given that the V122I TTR variant is present in up to 4% of Black individuals, who face persistent structural barriers that contribute to underdiagnosis and underrepresentation in genetic testing programs.

### 9.2 Limitations

There are several limitations that are apparent in this study. First, the possibility of recall bias due to participants' reflection on past experiences cannot be eliminated. Second, this study included a small number of patients from a single referral center, which limits transferability. Our center serves as a tertiary referral hospital and includes patients from neighboring states, reflecting a range of referring health systems and care contexts. However, participants were predominantly White, educated, and had baseline familiarity with genetic concepts, limiting the demographic diversity of the sample. In addition, there were only 2 participants who had a positive V122I TTR genetic variant, which is the most common in the United States. Furthermore, all patients have been previously diagnosed and can access specialized care. These findings also may not be applicable to other rare diseases that do not have treatments available like ATTR. Importantly, even with this relatively selective and engaged sample, participants demonstrated limited understanding of the roles of genetic counselors and substantial challenges with cascade testing communication. This suggests that these challenges may be exacerbated in more general or resource‐limited populations. Third, most participants had not received genetic counseling, limiting our findings primarily to the early phases of genetics engagement, when perceptions and referral decisions are formed. Fourth, although patients were self‐selected to participate in this study, they were recruited by their treating cardiologist. These selection biases may limit the range and authenticity of participant responses, potentially distorting our ability to fully capture the experiences of individuals in this context. Finally, while consistency was supported by collaborative coding in a subset of transcripts, recontact to provide participant feedback was not performed.

## 10 CONCLUSIONS

This qualitative study reveals gaps in patient understanding regarding genetic counselors' roles and the dependence of patient engagement on the strength of cardiovascular provider recommendation. Although only a minority of participants in our study had prior experience with a genetic counselor, those who did viewed the experience as helpful, especially with family member communication for cascade testing, and highlighted the need for counselors with cardiac‐specific expertise. These insights are essential for informing future implementation of science‐based strategies to enhance integration of genetic counselors and cascade testing within cardiovascular care.

## AUTHOR CONTRIBUTIONS

**Quan M. Bui:** Conceptualization; data curation; formal analysis; funding acquisition; investigation; methodology; validation; writing – original draft; writing – review and editing.

## CONFLICT OF INTEREST STATEMENT

Quan Bui is a recipient of the Janice Wiesman Young Investigator Grant (Ionis Pharmaceuticals) and the AHA Career Development Award (24CDA1272533). Larry Allen has received consulting fees from ACI Clinical, Quidel, and UpToDate, and grant funding from the NIH and PCORI. Marcus Urey has served on the advisory board and has received speaker fees from Alnylam, AstraZeneca, and BridgeBio. Marcus Urey also reports conflicts with Pfizer and has received research support for clinical trials from Ionis Pharmaceuticals and Alexion. None of the other authors has a financial relationship with a commercial entity that has an interest in the subject of the presented manuscript or other conflicts of interest to disclose.

## ETHICS STATEMENT

Human studies and informed consent: The study was approved by the UC San Diego Institutional Review Board and received appropriate ethical oversight. All participants provided written consent before enrollment into the study.

Animal studies: This research did not involve the use of animals.

## Data Availability

The data that support the findings of this study are available on request from the corresponding author. The data are not publicly available due to privacy or ethical restrictions.

## APPENDIX 1 INTERVIEW GUIDE

### PART 1: PATIENT DISEASE HISTORY

*Next, I will ask you some questions about your heart condition*.

- Can you tell me when you were first diagnosed with your heart condition?
  ⚬ What is your understanding of your illness? How would you describe your condition to a stranger?
  ⚬ How confident do you feel about your understanding of your heart condition?
  ⚬ Are you satisfied with the current work‐up and treatment for your heart condition?
  ⚬ Are you aware that there are genetic causes of heart disease?
- Has your medical provider ever discussed genetic testing as part of the work‐up of your heart condition?
  ⚬ *If yes*, please explain what you understand about genetic testing
    ▪ Describe the process of genetic testing.
    ▪ What is your understanding of the benefits of genetic testing?
    ▪ How might genetic testing be useful in identifying additional family members at risk?
    ▪ What are some of the treatment options available for individuals diagnosed with a genetic heart condition?
    ▪ What are the limitations and risks of genetic testing?
  ⚬ *If no*, how interested would you be in receiving more information about genetic testing related to your heart condition?
    ▪ What information do you wish you could know?

### PART 2: GENETIC TESTING AND COUNSELING

*Now, I will ask you some detailed questions about genetic testing and counseling*

- Tell me about your decision‐making process for genetic testing
  ⚬ How did (would) you come to your decision?
  ⚬ Who helped (would help you) with making your decision? (*Probe – did you have any family members or friends or clinical care team help?)
  ⚬ What information do you wish you had known when making the decision?
  ⚬ Were you shown any educational tools or decision aids to help you in the decision‐making process?
    ▪ If yes, what were they? How did you feel about them? Did they help you make your decision?
  ⚬ Do you wish you had more information? What type?
- How did you feel during the decision‐making process?
- What were the positive parts of the decision‐making process?
- What were the challenging parts of the decision‐making process?
- How did you feel about the explanations you received regarding the risks and benefits of genetic testing? (*Probe – did you feel that you were adequately informed?)
- What are your thoughts on the informed consent process you experienced related to genetic testing?
- Did you make your decision on the spot, or did you take additional time?
- Were there any things that surprised you about the genetic testing process?

*[If patient declined genetic testing]*

- What were the top three reasons that influenced your decision to decline genetic testing?
- How did potential effects on your family influence your decision regarding genetic testing?
- What concerns did you have about potential insurance and workplace discrimination risks related to genetic testing?
- How were your family members approached for clinical screening after you declined genetic testing?

*[If patient received genetic testing]*

- How did your medical provider discuss results of genetic testing with you? In‐person visit or over the phone/Zoom? Were you satisfied or did you want more information? Did you use the internet/Google search?
- How would you describe your medical provider's understanding of genetics and genetic testing?
- How did your medical provider support communication with your family members regarding genetic testing? Did he or she provide you with a letter to share with them?
- In what ways did your medical provider offer psychosocial support to you and family members? Did you find it sufficient?

*[Genetic counseling]*

- How was a genetic counselor involved in your genetic testing process? Before/after testing or both?
- How did your medical provider approach the topic of referring you to a genetic counselor?
- How did your medical provider explain the role of a genetic counselor?
- What importance did your medical provider place on seeing a genetic counselor, and what benefits were discussed with you?
- Did you end up seeing a genetic counselor?
  ⚬ *If yes*, did you find it helpful/useful?
    ▪ How did the information provided by your genetic counselor compare to that provided by your medical provider? Please elaborate
    ▪ How did your genetic counselor's interpretation of your genetic testing results compare to that of your medical provider?
    ▪ In what ways did your genetic counselor assist with cascade testing for your family members (if indicated)?
    ▪ How did you feel about the psychosocial support provided by your genetic counselor for you and your family members?
    ▪ Was your visit with the genetic counselor in‐person or via telehealth? How did that impact your experience?
    ▪ Do you think having a genetic counselor in the same clinic as your medical provider would be helpful? Please elaborate. (*Probe: would you be more likely to see a genetic counselor?)
- How do you feel about your decision to undergo genetic testing? How did the results and implications of genetic testing compare to your expectations?
